# Supplementary material for: Application of Biopsy Samples Used for Helicobacter pylori Urease Test to Predict Epstein–Barr Virus-Associated Cancer
Source: Microorganisms. 2020 Jun 18;8(6):923. doi: 10.3390/microorganisms8060923 (PMC7355529; doi:10.3390/microorganisms8060923)
Supplement: Supplementary file 1 [file microorganisms-08-00923-s001.pdf]

Supplementary figures

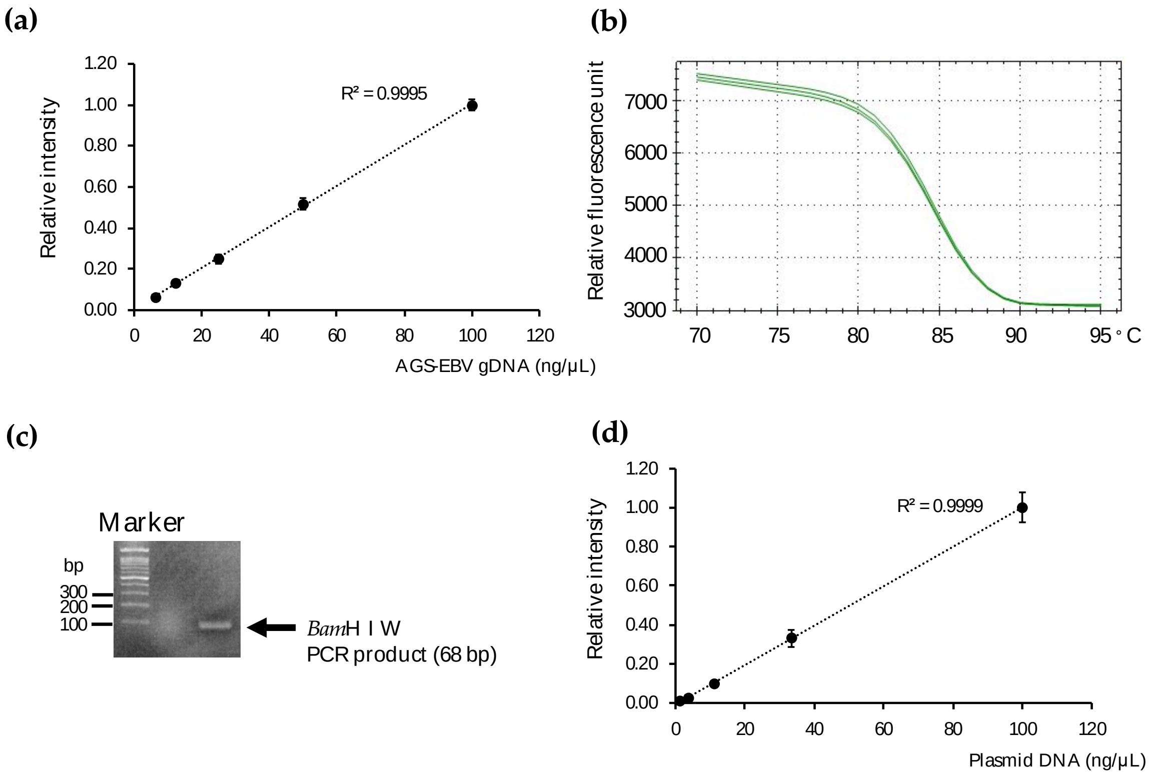

Figure S1. Evaluation of *Bam*H I W specific qRT-PCR (a) A calibration curve for *BamHI* W specific qRT-PCR using genomic DNA isolated from AGS-EBV cells as a template. The fluorescence intensity amplified from 100 ng of AGS-EBV cell DNA was defined as 1. n = 3. (b) Measurement of melting temperature of *BamHI* W PCR primer. n = 3. (c) Electrophoresis of PCR products. (d) A calibration curve for *BamHI* W specific qRT-PCR using *BamHI* W plasmid DNA as a template. The fluorescence intensity amplified from 100 ng of *BamHI* W plasmid DNA was defined as 1. n = 3.
